# Professionals' perspectives on the challenges of implementing digital solutions in rehabilitation settings in Rwanda

**DOI:** 10.3389/fdgth.2025.1489288

**Published:** 2025-08-14

**Authors:** Kaisa Jokinen, Michael Oduor, Gerard Urimubenshi, Juliette Gasana, Jean Damascene Bigirimana, David K. Tumusiime, Eeva Aartolahti, Kari-Pekka Murtonen, Katariina Korniloff

**Affiliations:** ^1^ Institute of Rehabilitation, Jamk University of Applied Sciences, Jyväskylä, Finland; ^2^ Department of Physiotherapy, School of Health Sciences, College of Medicine and Health Sciences, University of Rwanda, Kigali, Rwanda; ^3^ The Regional Centre of Excellence in Biomedical Engineering and eHealth, University of Rwanda, Kigali, Rwanda

**Keywords:** digital rehabilitation, digitalization, rehabilitation, Rwanda, socioeconomic factors, digital technologies

## Abstract

The need for rehabilitation is unmet, especially in low- and middle-income countries such as Rwanda. Digital rehabilitation offers significant potential for delivering rehabilitation services in low-resource settings, and this study examines the challenges affecting the digitalization of rehabilitation. Semi-structured interviews and a survey were conducted in Rwanda with rehabilitation professionals to collect data for two different projects. The different datasets were analyzed using thematic analysis and inductive content analysis. As a result, three main concepts were formed: context-related factors, individual-related factors, and technology-related factors. Results suggest that the challenges in implementing digital solutions in rehabilitation settings in Rwanda encompass various domains, including socioeconomic factors, infrastructure, digital competency, regulatory frameworks, and user-related factors. In conclusion, because of multifaceted challenges, systemic-level change is needed to realize the potential of the digitalization of rehabilitation and other health care services in Rwanda.

## 1 Introduction

Rehabilitation, as defined by the World Health Organization (WHO), consists of targeted interventions designed to improve functional performance and reduce disability in people whose health conditions affect how they interact with their environment, focusing on personal capabilities and environmental circumstances (1). It is estimated that globally around 2.4 billion people would benefit from rehabilitation due to their health conditions. This need is expected to increase due to various reasons, such as changes in population characteristics and increasing number of chronic diseases. However, access to rehabilitation services remains limited, especially in low- and middle-income countries (LMIC), where over 50% of the population is unable to access rehabilitation services (2).

The integration of digital technologies in rehabilitation settings holds significant promise for enhancing healthcare delivery, particularly in low-resource settings like Rwanda, where there is a lack of professionals (2). Digital health technologies have the potential to improve access to rehabilitation, patient outcomes, and resource utilization (3). Digital rehabilitation (DR) is an umbrella term for rehabilitation interventions using digital solutions. It is a multidisciplinary term encompassing every stage of the rehabilitation process, including prevention. Remote rehabilitation applications, telerehabilitation solutions, or remote monitoring methods are examples of interventions that can be used in DR settings (4).

Despite this potential, the implementation of DR in LMICs faces numerous challenges, including weak health systems, limited infrastructure, and insufficient digital literacy (5, 6). These barriers are often more pronounced in rural areas where health care services are already scarce (7, 8). Moreover, policy and regulatory frameworks in many LMICs remain underdeveloped, further hindering the integration of digital solutions into healthcare systems (9). Rwanda serves as a valuable setting for studying these problems. As a low-income country in Eastern Africa, Rwanda has made notable progress in digital transformation and healthcare system strengthening (10).

Rwanda has prioritized digital health as part of its national developing strategy. However, like many LMICs, it continues to face systemic and socioeconomic challenges that complicate the implementation of DR services (11). Currently, there are no rehabilitation professionals working at the primary health care level in Rwanda; the district hospitals are the first institutions to host a limited number of physiotherapists. The number of physiotherapists per 10,000 people is 0.29, while for occupational therapists it is 0.02 and for prosthetics and orthotics professionals 0.05 (12). These workforce limitations further highlight the need for scalable and accessible DR solutions.

While existing literature highlights structural and infrastructural barriers to DR in LMICs, there is a notable gap in understanding how these challenges are perceived by rehabilitation professionals. Their insights are crucial for identifying context-specific challenges and opportunities for digital integration.

Therefore, the purpose of this study is to explore the socioeconomic and systemic challenges that professionals encounter when implementing DR solutions in Rwanda. By addressing this gap, the study aims to inform strategies that support the effective integration of digital technologies into rehabilitation services in similar low-resource contexts. We have used two separate datasets to ensure a sufficiently comprehensive view of the national situation in Rwanda from rehabilitation professionals' perspective and to confirm data saturation.

## 2 Participants and design

The data for this study was collected through two projects that aimed to promote the implementation of and higher education about DR in East Africa. The DIRECT project (Co-innovation of digital rehabilitation in the global marketplace) aims to improve access to rehabilitation services using evidence-based digital technologies. The RADIC project (Rehabilitation for All through Digital Innovation and new Competences) aims to enhance the capacity of higher education institutions (HEIs) in East Africa to address the growing need for digital competence among professionals in rehabilitation via curriculum renewal and new teaching methods.

All the participants in both projects signed an informed consent form. Ethical clearance for the DIRECT and RADIC research activities was respectively provided by the Rwanda National Ethics Committee (RNEC) and the Institutional Review Board (IRB) of the University of Rwanda-College of Medicine and Health Sciences (UR-CMHS).

To achieve our goals, qualitative methods and the interpretative paradigm were adopted. The choice of the paradigm was based on the interest to gain a deeper, individual-level understanding of the rehabilitation situation in Rwanda, in addition to needs and challenges related to DR.

The DIRECT data is based on six individual interviews conducted in September 2022 that were a follow-up to a study examining Rwandan rehabilitation professionals' experiences of a DR application. The study purposefully included the most active users of the DR application among those readily available. Participants' (Table 1) experiences were recorded using a semi-structured interview guide encompassing open-ended questions around use of technology and barriers and enablers to accessing rehabilitation in Rwanda. The interviews were conducted in the language of the participants' choice if they could not communicate in English.

**Table 1:** Demographic characteristics of DIRECT study participants, *N* = 6.

Variable	Representation	*n*
Education	Technical/vocational qualification or equivalent (Diploma)	1
	Bachelor's degree or equivalent (BSc)	3
	Professional degree, master's degree, or equivalent	2
Profession	Physiotherapist	5
	Assistant Physiotherapist	1
Work experience (years)	5–10	2
	>10	4
Self-reported technology experience	Very little	1
	Average	2
	Quite extensive	1
	Very extensive	2
Self-reported digital health technology experience	Average	3
	Quite extensive	2
	Very extensive	1

For RADIC, a survey with open-ended questions was chosen to collect the views from the rehabilitation professionals from a larger geographic area (Rwanda, Tanzania, and Kenya). The anonymous survey conducted in January-March 2024 included four demographic questions and seven open-ended questions. The participants (Table 2) were recruited via mailing lists, discussion groups, and academic networks. The survey aimed to collect the views of rehabilitation professionals on the needed digital competencies and challenges they face when using or considering the use of digital solutions in rehabilitation in their work. For this study, only the data acquired from Rwanda was used.

**Table 2:** Demographic characteristics of the RADIC survey participants, *N* = 45.

Variable	Representation	*n*
Occupation	Physiotherapist	33
	Occupational therapist	11
	Prosthetics & orthotics	1
Work experience (Years)	<5	13
	5–10	22
	>10	10
Level of health care situated	National referral hospital	9
	Regional hospital	1
	District hospital	1
	Private health care service	6
	Dispensaries/clinics/health centers	2
	Other community-based facilities	5
	Other	12

### 2.1 Data analysis

The data analysis was conducted manually using MS Office tools (Word and Excel) and, additionally, a digital whiteboard tool in the concepting phase of RADIC data. The data was pseudonymized at the start of the analysis phase. The process was initialized by defining the research question, “What socioeconomic challenges affect the application of digital solutions in rehabilitation settings in Rwanda?” However, during the analysis process, the scope of the question was seen as too narrow, and the question was broadened from “socioeconomic challenges” to “challenges.” In reporting of the results, abbreviations D(No.) refer to citations from the DIRECT and R(No.) for the citations from the RADIC.

The DIRECT data was analyzed by one researcher (MO) using thematic analysis guided by the process illustrated by Brown and Clarke (13). Thematic analysis was chosen to understand the professionals' experiences, ideas, and perceptions of DR. The process comprises developing initial coding ideas from the data, coding and matching codes to extracts, and sorting the codes into potential themes. Then the relationships between the themes are analyzed, reviewing and refining them, and finally all codes are categorized according to the final formed themes (13). The analysis was discussed with other researchers (EA, KK) to validate the results.

The RADIC data was analyzed using inductive content analysis by one researcher (KJ) utilizing the process described by Kyngäs et al. (14) The analysis method was chosen to describe naturally emerging themes and patterns arising from the participants' responses. The data was read through multiple times, and the analysis unit was determined to be “statement”. A total of 76 statements were found to address the research question. The statements were reduced to a simpler form and grouped to create 35 sub-concepts. These sub-concepts were then abstracted to become ten concepts, which in turn were shared to form three main concepts and one combining concept. The analysis results were discussed with another researcher (KK) to validate the process.

**Figure 1:**
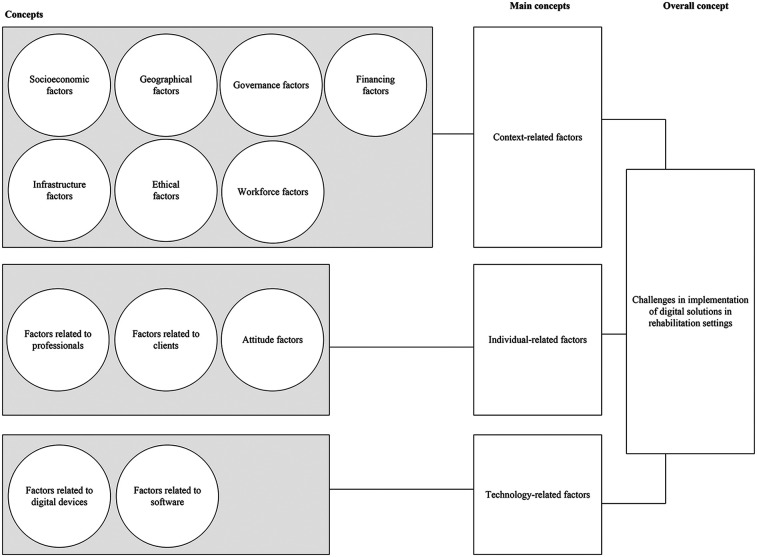
The concepts summarizing the challenges that affect the use of digital rehabilitation in Rwanda.

After analyzing both data, the themes and concepts were compared and combined by one researcher (KJ), and the result was discussed with other researchers (MO and KK) to reach a common understanding. The summary of the combined analysis is presented in Figure 1.

## 3 Results

This section presents the combined findings from the two projects' qualitative analyses. The findings on challenges to implementing DR are categorized into three main concepts: contextual, individual, and technological factors. Of these main concepts, the contextual factors are the most important, as they influence the effect of the latter two.

### 3.1 Context-related factors

Context-related factors are those related to the context in which individuals live. There are factors that individuals can change and influence, while others are determined by the system.

“The issue is the geographic location, the issues of health public policy, the issue of under income countries in Africa, education factors, social economy factors, the issues of health equity.”—R34

#### 3.1.1 Factors related to digital devices

Illiteracy, also linked with unequal educational possibilities, was often mentioned. Different age groups, especially elderly citizens, are in an unequal position. Some service users are poor and unemployed. Poor financial status greatly affects the possibilities of accessing rehabilitation services, including digital processes. The socioeconomic factors arose clearly from both data.

“Some of our clients do not have knowledge and are non-educated, others they don't have smart phones.”—R26

“Generally, I can say that accessibility of rehabilitation service in Rwanda is not very easy for many people, especially those living in rural areas who cannot afford it.”—D1

#### 3.1.2 Geographical factors

There is inequality between urban and rural settings. The geographical context in Rwanda is challenging, and people in need of rehabilitation often live far from health facilities that have rehabilitation services, which prevents them from seeking care. Especially in DIRECT data, the distances and geographic inequalities were addressed more in depth, while in RADIC data, the focus was more on the availability of reliable connections and infrastructure in the rural areas.

“The patients still don't go there (when referred) because it is very far from their places and long appointments.”—D3

“A patient in a rural area struggle with a telehealth session due to unreliable internet, limiting the effectiveness of remote rehabilitation.”—R25

#### 3.1.3 Governance factors

Overall, Rwanda's status of a low-income country influences health care policies. There are no specific policies or legal frameworks for rehabilitation. A need for supportive context-specific policies to regulate the implementation and use of DR services was identified in both data. Unavailable policies are closely linked to financing factors, as this influences what the insurances cover.

“No specific policy from policymakers regarding rehabilitation. So far they did already what we call need assessment, and the report is out now and it tackled each and every angle so that it gives hope maybe in the future it will be considered in strategic plan of different institutions to give a line in terms of rehabilitation”—D4

“Unavailable policy and country guidelines for digital rehabilitation.”—R36

#### 3.1.4 Infrastructure factors

Challenges with electricity as well as the availability, cost, and reliability of internet connectivity were identified as challenges for all professionals and clients. In addition, it was mentioned that the geographical location affects the situation related to infrastructure, as the rural areas are in an unequal situation compared to urban.

“Lack of sufficient technological infrastructure, poor internet connectivity to both sides–”—R19

“Poor and unstable internet, inadequate infrastructure.”—R36

“For someone who has a disability and wants to go to a district hospital in rural areas where there is no transport and sometimes, they have to carry children; It will become more difficult to get that special healthcare.”—D4

#### 3.1.5 Ethical factors

Sharing the available devices is one way of mitigating the lack of own devices, but it endangers confidentiality and privacy. Ensuring data security might become an issue. One critical matter is how the high quality of DR services is ensured with many challenges posed by socioeconomic issues. In RADIC data, it was also noted that professionals need more information and skills related to privacy issues.

“Challenge is most of client doesnt have appropriate telephone for video call, or unable to write sms, they can use another person, but this is not right in term of confidentiality.”—R10

“(Professionals need) ability to maintain and apply privacy policies concerning with digital rehabilitation or tele-rehabilitation.”—R26

#### 3.1.6 Workforce factors

Issues with the workforce arose more clearly from DIRECT data. Public health institutions lack multidisciplinary rehabilitation professionals, and their distribution across different healthcare levels is uneven. Professionals are mostly available in urban areas. In RADIC data, this was discussed via how digital solutions could enhance the accessibility to limited rehabilitation services.

“Another issue is the strategy of the government; on their structure they only allow 2 physiotherapists at district hospitals–”—D1

“To be honest it is not accessible especially in provinces…But here in Kigali they (service users) understand rehabilitation services and come for therapy even though the numbers are still low… In provincial and district hospitals they also have a low number of therapists which is also a challenge for people living in rural areas.”—D2

#### 3.1.7 Financing factors

The need to develop, reform, and enforce supportive health legislation and policies that mitigate challenges to accessing services was also highlighted. Including increased funding for rehabilitation, assistive devices, and their different delivery mechanisms to address affordability issues. Financing was discussed only in DIRECT data to this extent.

“Now it’s (the cost) quite reduced because we are using community-based health insurance, but it is not covering all the services of Rehabilitation like in P&O you may go there but the insurance will not cover prosthesis–”—D3

“And most of the population for example 85% of the Rwandan population are using the CBHI (Community Based Health Insurance) and they are not able to pay for these services… because the insurance can not pay for them without a medical prescription. That’s one of the challenges hindering the accessibility of rehabilitation services.”—D5

### 3.2 Individual-related factors

Unlike contextual factors, individual factors can be changed and influenced with the right socioeconomic conditions. As the rehabilitation process is a process between a professional and a client, the individual-related factors were divided accordingly.

#### 3.2.1 Factors related to clients

Individual factors are closely linked to context-related factors. Clients often have limited ability to use technologies and limited digital competency due to lack of exposure. In addition, clients may not possess knowledge of the possibilities of applying technological solutions. Limited language proficiency hinders the effective use of digital solutions in rehabilitation. Barriers related to clients were identified in both data.

“Internet connections and patients’ capability of using digital technology.”—R3

“Patients are not skilled in digital materials and some of them lack those materials.”—R40

“See technology is really still new to many people and sometimes we have patients that don't have smartphones and are not well educated to use[the digital rehabilitation tool].”—D4

#### 3.2.2 Factors related to professionals

The professionals should be proficient in digital technology, as they may be required to assist clients with technical issues. This is not, however, the case. Poor digital skills and inadequate training were identified as challenges, and the need for continuous training was highlighted. This may not be achieved due to socioeconomic challenges. In DIRECT data, the focus was more directed towards the number of professionals rather than their digital competence.

“We cannot treat through digital rehab if we do not have idea about how to use these machines appropriately”—R22

“They (professionals) need to be trained about how to use it and this will improve the effectiveness of treatment for the patients.”—R19

#### 3.2.3 Attitude factors

Attitude factors derive from a lack of knowledge and from the distrust of the effectiveness of digital solutions in rehabilitation. People are accustomed to traditional methods and, for instance, it may be challenging for clients to follow technology-based rehabilitation programs. Attitudes and prejudices towards digital technologies rose from RADIC data; in DIRECT data, attitudes were more discussed related to seeking rehabilitation services in general.

“Client awareness of their need to participate in the therapy. Example, I gave a lot of the therapeutic activities to do at home as I called to check up implementation they all didn't apply, with a lot of excuses I can't do it like you, have no time, don't think I need a lot of therapy as long as I attended with you face to face.”—R39

“Low level of understanding and trust of effectiveness of digital solution for patient side.”—R29

### 3.3 Technology-related factors

When discussing the socioeconomic challenges in DR, it is only natural that digital technologies play a role. However, it is important to realize that when discussing technology, it is not solely about the devices but also the software.

#### 3.3.1 Factors related to digital devices

Most often, it was stated that the clients do not have access to the necessary devices partly due to cost and availability issues. Generally, lack of electronic devices and IT equipment was mentioned, as was the general inequity in access to technology. Lack of suitable devices was identified in both data.

“–lack of adequate digital materials like computers, internet disruption when therapeutic session is going on, and some clients did not have smart phone.”—R38

“Internet connection issues, limited knowledge of the service users/patients, lack of appropriate device to use (laptop with good camera).”—R27

“The parents have been saying we don't have technology devices (smartphones), we don't have airtime, we don’t have this and this…”—D2

#### 3.3.2 Factors related to software

The cost of software and licenses could be a significant hurdle. It was also mentioned that not everyone is able to have access to digitalized materials. The accessibility of software is another important factor, as the use of international languages, such as English, can create barriers for uneducated users with limited resources.

“Most of the challenges we faced are our patients, some are not able to give us feedback about home therapy exercises through available rehabilitation technological tool because all of them are in international languages.”—R31

“Not everyone understands how to use […]. Sometimes it is difficult for them to understand the language and I cannot translate for them apart from the explanations I can give them hoping that they will remember.”—D6

## 4 Discussion

This study aimed to explore the challenges that impact the utilization of digital solutions from rehabilitation professionals' perspectives in rehabilitation settings in Rwanda. Specifically, barriers to accessing rehabilitation services using digital solutions were examined. Three main concepts were identified: contextual, individual, and technological factors. These factors are comparable to those presented in the FITTE framework, which describes the factors affecting the use of technology in clinical settings, these being individual, task, technology, and environment (15). By using two different datasets, the main concepts could be validated, and all relevant sub-concepts and saturation confirmed, as both data brought to the analysis something that the other was lacking. For instance, the DIRECT data was more focused on financing and availability of the workforce, and the RADIC data on attitudes, skills, and competencies related to digitalization.

The challenges in implementing digital rehabilitation in Rwanda, according to rehabilitation professionals in this study, encompass various context-related issues, including infrastructure, regulatory frameworks, and socioeconomic challenges. This is in accordance with previous research (5, 6, 8). For example, Rwanda's healthcare infrastructure, particularly in rural areas, still faces significant gaps in terms of access to reliable electricity, internet connectivity, and adequate facilities. Even though Rwanda is situated in the good middle-cast of African countries in ICT (Information and Communication Technology) Development Index (IDI) classification with rapidly improving results (16), the lack of robust infrastructure still poses a major barrier to the widespread adoption of DR technologies.

In contextual factors, many underlying factors, such as gaps in health policies supporting the delivery of sufficient rehabilitation services and financing factors, are closely related as identified in this study, and it is often hard to separate one from another. Still, professionals have a positive outlook on the development of rehabilitation services in Rwanda. However, they also acknowledge that there is plenty of work to be done. The absence of policies governing the use of digital health technologies poses challenges related to the implementation of DR interventions and hampers the scaling up of DR initiatives. The need for policies to support the use of DR is also acknowledged by others (5, 9, 17).

According to our results, socioeconomic factors, such as poverty, illiteracy, and unequal opportunities with technologies, are barriers to accessing DR services in Rwanda. These factors could be counted under many of the concepts in our results, as the socioeconomic factors seem to be entwined. For instance, the reasons for poverty may be due to either geographical location where the opportunities for employment are scarce or sudden sickness resulting in out-of-pocket costs if the social security system does not offer support. As the results are obtained from solely Rwandan professionals, they are not directly transferable to other low- and middle-income countries, but for similar settings, the results may provide insights into the obstacles that digitalization of rehabilitation may encounter. Similar context-related challenges were identified in recent literature review addressing rural areas of Africa, in which, e.g., poor infrastructure, geographic constraints, and digital illiteracy were identified as major barriers (18) and similar challenges were identified also in Vietnam (19).

Individual-related factors in our results are focused rather on the skills and competences of the clients and professionals, as the socioeconomic traits are included under contextual factors. The importance of digital competence on both sides is proven in prior research focusing on low- and middle-income countries (19, 20). In addition, attitudes are counted as individual-level factors, even though the cultural norms and beliefs might influence them. In recent study, it was noted that experienced negative emotions indicated that the professional is unlikely to use technologies further (21).

When exploring the concept of DR (4), it is natural that technology-related factors would be highlighted as a main concept. Technology can act as a powerful facilitator to equal access to rehabilitation, but it needs to be understood that in many contexts it might be a barrier to, or at least an obstacle that needs to be solved. Affordability, availability, and accessibility of necessary technology are crucial components in ensuring high-quality digital services. This has also been noted in previous research focusing on low- and middle-income countries' contexts (6). Scaling the digital services to answer the needs and opportunities of the clients is of utmost importance when planning the initiatives in the future.

Effective use of ICT to improve health outcomes and combat diseases among marginalized and isolated populations is a significant challenge for developing countries. This is an area where the potential for effective use of the full range of ICT is immense (22). Additionally, attitudes and beliefs around technologies influence the acceptance and utilization of DR services in Rwanda (21). Consideration of cultural norms and preferences, as well as pre-existing beliefs about the effectiveness of digital interventions, is crucial for designing interventions that are culturally appropriate and acceptable to the target population.

### 4.1 Limitations

Based on our results, which focus on rehabilitation, we cannot make conclusions about all sectors of health care, but these results may be partly applicable to the utilization of digital health solutions in similar contexts. In relation to data acquired via RADIC activities, it is worth noting that the data was collected using digital means. This may have affected the response rate and excluded respondents who are unwilling or unable to use digital methods.

## 5 Conclusion

This study explored various contextual, individual, and technology-related challenges that professionals face when implementing digital rehabilitation solutions in Rwanda. The results suggest that these challenges span multiple domains, including infrastructure and geography, regulatory frameworks, technological limitations, and individual-related factors. In conclusion, due to multifaceted challenges, systemic-level change is needed to fully reach the potential of the digitalization of rehabilitation and other health care services. Further research, including gathering the insights of the clients, is needed to support the utilization of context-scaled digital innovations in the field of health care in Rwanda.

## Data Availability

The raw data supporting the conclusions of this article will be made available by the authors, without undue reservation.
